# Space Confinement to Regulate Ultrafine CoPt Nanoalloy for Reliable Oxygen Reduction Reaction Catalyst in PEMFC

**DOI:** 10.1002/advs.202206062

**Published:** 2023-05-10

**Authors:** Weikang Zhu, Yabiao Pei, Haotian Liu, Runfei Yue, Shilin Ling, Junfeng Zhang, Xin Liu, Yan Yin, Michael D. Guiver

**Affiliations:** ^1^ State Key Laboratory of Engines School of Mechanical Engineering Tianjin University Tianjin 300072 P. R. China; ^2^ National Industry‐Education Integration Platform of Energy Storage Tianjin University Tianjin 300072 P. R. China

**Keywords:** CoPt nanoalloy, oxygen reduction reaction, proton exchange membrane fuel cell, PtM catalyst, space‐confined effect

## Abstract

A Co‐based zeolitic imidazolate framework (ZIF‐67) derived catalyst with ultrafine CoPt nanoalloy particles is designed via a two‐step space confinement method, to achieve a robust oxygen reduction reaction (ORR) performance for proton exchange membrane fuel cell (PEMFC). The core–shell structure of ZIF‐67 (core) and SiO_2_ (shell) is carefully adjusted to inhibit the agglomeration of Co nanoparticles. In the subsequent adsorption−annealing process, the in situ formed graphene shell on the surface of Co nanoparticles further protects metal particles from coalescence, leading to the ultrafine CoPt nanoalloy (average diameter is 2.61 nm). Benefitting from the high utilization of Pt metal, the mass activity of CoPt nanoalloy catalyst reaches 681.8 mA mg_Pt_^−1^ at 0.9 V versus RHE according to the rotating disk electrode test in 0.1 m HClO_4_ solution. The CoPt nanoalloy‐based PEMFC provides a high maximum power density of 2.22 W cm^−2^ (H_2_/O_2_) and 0.923 W cm^−2^ (H_2_/air). Simultaneously, it shows good stability in the long‐time dynamic test at low humidity, due to the robust CoPt@graphene core–shell nanostructure. This work provides a viable strategy for designing Pt‐based nanoalloy catalysts with ultrafine metal particles and high stability.

## Graphical abstract

A two‐step space confinement method is proposed to refine the size of the CoPt nanoalloy during zeolitic imidazolate framework (ZIF) derived catalyst synthesis. Due to the well‐controlled nanoparticle size and graphene shell protection, the mass activity of the CoPt nanoalloy catalyst reaches 681.8 mA mg_Pt_^‐1^ at 0.9 V versus RHE with excellent stability during dynamic fuel cell tests.

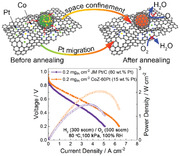

## 1 Introduction

Proton exchange membrane fuel cells (PEMFCs), as promising energy conversion devices, are used for multiple purposes such as vehicle and backup power. However, the development of fuel cells is hampered by the cost and scarcity of Pt‐group metals. The shape, size, and surface distribution of Pt nanoparticles, as well as physicochemical properties contribute to the electrocatalytic activity and durability during PEMFC application.^[^ ^1^ ^]^ The slow oxygen reduction reaction (ORR) kinetics and acid environment in the cathode requires the Pt‐based catalyst to have high intrinsic activity and good stability. Alloys of two or more metals are considered as effective and convenient ways to enhance the electrocatalytic activity of ORR.^[^ ^2^ ^]^ Based on density functional theory (DFT) calculations, various Pt‐transition metal (PtM) alloy catalysts^[^ ^3^ ^]^ have been developed, such as NiPt,^[^ ^4^ ^]^ CoPt,^[^ ^4b^ ^]^ and FePt.^[^ ^5^ ^]^ Different from cubic Pt_3_M nanoalloy, tetragonal PtM (such as FePt and CoPt) shows the strong coupling of M (3d) and Pt (5d) atomic orbitals along the crystallographic *c* direction, leading to high stability against acid etching.^[^ ^6^ ^]^ To obtain the CoPt catalyst efficiently, a deposition‐precipitation method was developed.^[^ ^7^ ^]^ Co and Pt (atomic ratio 95:5) were co‐deposited on commercial carbon black (XC‐72) by low‐temperature heat treatment under different atmospheres (H_2_ or CO/Ar) to enhance electron relocation and localization around Pt atoms from their neighbors. The annealing of CoPt in CO form a negative charge field around Pt atom, thus enhancing the mass activity (MA) of Pt by 4.9‐fold compared with commercial Pt/C. To further control the morphology of Pt‐based nanoalloy, an organic phase synthesis method was developed. CoPt and FePt nanowires were obtained by the decomposition of metal pentacarbonyl and reduction of platinum acetylacetonate in a solution of 1‐octadecene and oleylamine.^[^ ^8^ ^]^ Similarly, assorted CoPt‐based nanoparticles,^[^ ^6^, ^9^ ^]^ nanocrystals,^[^ ^10^ ^]^ nanodendrite, and nanoframe^[^ ^11^ ^]^ were synthesized by similar method. The obtained PtM nanoalloy particles are typically loaded on carbon supports for fuel cell application. Due to the weak physicochemical interaction between carbon support and ex‐situ grown PtM nanoalloy, poor stability, and considerable contact resistance are inescapable. During the PEMFC application, the unstable connection may accelerate the decay of fuel cell performance.

To improve the stability of ORR catalysts, the properties of carbon supports are drawing increasing attention, which requires maximizing specific surface area for high metal content, good conductivity for fast electron transport, the rational pore size distribution for efficient gas/water diffusion, as well as good physicochemical stability.^[^ ^12^ ^]^ Metal–organic frameworks (MOFs) with intrinsically isolated metal nodes and N‐containing ligands are considered as one of the most promising candidates for Pt‐based and Pt‐free catalyst synthesis.^[^ ^13^ ^]^ Among them, Zeolitic imidazolate framework (ZIF) derived carbon catalysts with abundant pores and stable structure shows excellent promise.^[^ ^14^ ^]^ The particle size of ZIF can be accurately adjusted by changing the reaction temperature,^[^ ^15^ ^]^ introducing triethylamine (TEA),^[^ ^16^ ^]^ changing solvent,^[^ ^17^ ^]^ etc. This provides the possibility for structural regulation of the catalyst layer during membrane electrode assembly (MEA) fabrication. Based on ZIF precursors, various PtM alloy catalysts have been designed and synthesized to optimize the active site structure and improve the ORR performance for PEMFC application. Recently, two differently structured active sites, i.e., ordered CoPt intermetallic nanoparticles and Pt single atom site, have been incorporated into ZIF‐derived catalyst.^[^ ^18^ ^]^ In situ electron microscopy confirms the annealing process can form a highly active CoPt nanoalloy. In addition, excess Pt atoms preferentially occupy under‐coordinated surface sites of PtM nanoalloy particles and form a Pt‐shell, which improves the stability in the acid environment.^[^ ^12^ ^]^ Combining the advantages of ZIF substrate and annealing treatment, a CoPt nanoalloy catalyst was synthesized based on Co/Zn‐ZIF derived carbon substrate.^[^ ^19^ ^]^ However, due to the severe agglomeration of Co nanoparticles during the substrate fabrication, the MA and fuel cell performance remains unsatisfactory. The SiO_2_ coating protection method has been exploited to restrict the free migration and aggregation of metal atoms during high‐temperature pyrolysis.^[^ ^20^ ^]^ With this knowledge, the Co particle size can be controlled by adjusting the ZIF precursor and SiO_2_ coating shell to provide a suitable Co‐based substrate for CoPt catalyst synthesis.

**Figure 1:**
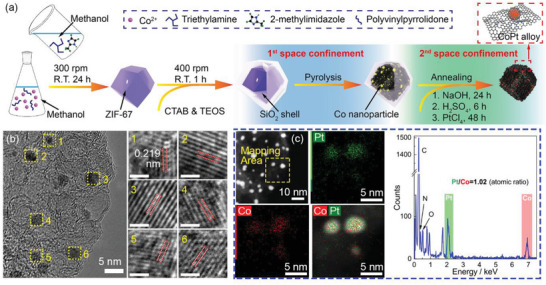
a) An illustration of the fabrication process of ZIF‐67‐derived CoPt nanoalloy catalyst. b) The high‐resolution TEM image of CoZ‐60Pt catalyst edge and corresponding lattice fringes. The scale bar represents 1 nm. c) The STEM element mapping and the corresponding elemental ratio of Pt and Co elements.

Herein, we report an ultrafine CoPt nanoalloy catalyst fabricated by a two‐step space confinement method, as shown in **Figure** **1**a. The ZIF‐67 particles are carefully adjusted by TEA and coated by SiO_2_ shell to obtain ultrafine Co nanoparticle carbon substrate. Simultaneously, a thin graphene shell is formed around Co nanoparticles, which can protect the CoPt nanoalloy from agglomeration in the following 900 °C annealing. Benefitting from the two‐step space confinement, the CoPt particle shows a size of <3 nm with high utilization of Pt atoms. Interestingly, the graphene shell originating from ZIF pyrolysis can also protect the CoPt nanoalloy during fuel cell long‐time stability test, exhibiting impressive durability.

## 2 Results and Discussion

### 2.1 Space Confinement Synthesis

A ZIF‐67‐derived carbon framework (embedded with Co nanoparticles) was obtained based on previous research^[^ ^21^ ^]^ and served as a substrate for the synthesis of CoPt nanoalloy. For the ZIF‐67 synthesis, TEA was added to reduce ZIF particle size, and tetraethyl orthosilicate (TEOS) served as a Si source to form SiO_2_ shell. First, the added amount of TEA was regulated to obtain a ZIF precursor with optimal particle size. For CoZ‐60TEA precursor, a 130 nm ZIF particle can be observed with an 8.5 nm shell in the transmission electron microscope (TEM, Figure S1, Supporting Information) image. The energy dispersive X‐ray spectroscopy (EDS) mapping analysis of Co and Si elements by scanning transmission electron microscopy (STEM) indicates the shell on the ZIF particle is constructed by SiO_2_ (denoted as ZIF‐60TEA@SiO_2_). With the increase of TEA amount from 0 to 80 µL during ZIF synthesis, the particle diameter of ZIF‐derived substrates decreases obviously from >500 nm for CoZ‐0TEA to 100 nm for CoZ‐80TEA (Figure S2, Supporting Information). However, the Co nanoparticle size shows a trend of first decreasing and then increasing. The STEM high‐angle annular dark field (HAADF) image of CoZ‐60TEA shows a large amount of ultrafine Co nanoparticles with the mean particle size (MPS) of only 2.35 nm (Figure S3, Supporting Information). The size of Co metal is influenced by the ZIF precursor diameter and SiO_2_ shell. Specifically, the SiO_2_ shell can impose restrictions on the migration of metal species in small ZIF particles during pyrolysis. Simultaneously, abundant N atoms and defects can anchor the metal nanoparticles, leading to smaller Co nanoparticles. As shown in the high‐resolution X‐ray photoelectron spectroscopy (XPS) of Co 2p (Figure S4, Supporting Information), Co^0^, Co^3+^ and Co^2+^ signals occur at 778.6, 779.8, and 783.1 eV, respectively.^[^ ^22^ ^]^ The dominant Co^2+^ and Co^3+^ species in CoZ‐60TEA indicate that a large amount of Co‐N_x_ species form during high‐temperature pyrolysis. The obvious Co^0^ peaks also imply the formation of Co nanoparticles in ZIF‐derived carbon substrates. The nitrogen adsorption‐desorption isotherms of different substrates were analyzed to investigate the porous structure. As shown in Figure S5 (Supporting Information), CoZ‐60TEA exhibits a large specific surface area of 712 m^2^ g^−1^. With the agglomeration of Co and the collapse of microporous structures, the specific surface area of other catalysts decreases significantly. To show the relationship between structure and electrochemical performance, all the ZIF‐67‐derived substrates were evaluated by RDE (in Figure S6, Supporting Information). Due to the co‐existence of smaller Co particles and Co‐N_x_ active sites, CoZ‐60TEA shows good ORR activity. The half‐wave potential (*E*_1/2_) reaches 0.74 V versus RHE in 0.1 m HClO_4_. The above result indicates that CoZ‐60TEA exhibits a suitable microstructure (smaller Co nanoparticles and abundant pores) for CoPt nanoalloy catalyst fabrication.

Based on the regulation of Co nanoparticle substrate, Pt element is introduced by mixing the obtained substrate with 2 mM PtCl_4_ solution, followed by annealing at 900 °C for 1 h. At the edge of the catalyst particle, metal nanoparticles with noticeable lattice fringes are observed in Figure 1b. In the enlarged panes on the right, the observed lattice distances are 0.219 nm, corresponding to the (111) facet of CoPt nanoalloy,^[^ ^6^, ^7^ ^]^ indicating the complete alloying of Co nanoparticles. To examine the elemental distribution and ratio of CoPt nanoalloy, STEM‐EDS mapping was carried out for CoZ‐60Pt catalyst. As shown in Figure 1c, both Co and Pt are principally distributed on the bright spots in the HAADF‐STEM image. The ratio of Co and Pt is close to 1, confirming the formation of CoPt nanoalloy. The STEM‐EDS analysis of a large‐scale sample indicates the presence of residual Si in CoZ‐60Pt catalyst (Figure S7, Supporting Information), which may improve the surface wettability of the catalyst during ORR process.^[^ ^23^ ^]^

### 2.2 Formation Mechanism

**Figure 2:**
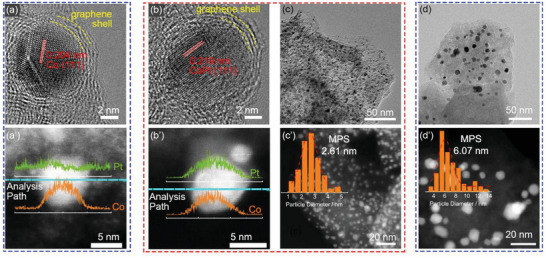
The high‐resolution TEM, HAADF‐STEM images and the corresponding Pt, Co element linear analysis curves of a) CoZ‐60Pt‐soak and b) CoZ‐60Pt. The metal particle size analysis of Pt‐based catalyst c) with or d) without the protection of graphene shell.

**Figure 3:**
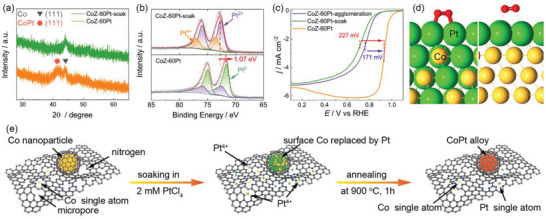
The a) XRD patterns, b) Pt 4f XPS spectra, and c) ORR LSV data of CoPt‐based catalysts. d) The atomic models of CoPt@Pt (left) and Co@Pt (right) active sites after geometry optimization via DFT calculation. e) The schematic diagram of the CoPt nanoalloy particle synthesis.

The formation mechanism of ultrafine CoPt nanoalloy was investigated by TEM images and element analysis before and after annealing (denoted as CoZ‐60Pt‐soak and CoZ‐60Pt, respectively). As shown in **Figure** **2**a, for CoZ‐60Pt‐soak, a spherical metal particle can be observed with a multilayer porous graphene shell wrapped on the surface. The lattice distance of the metal particle is 0.204 nm, attributed to the (111) facet of Co nanoparticles.^[^ ^21a^ ^]^ The corresponding linear elemental analysis (Figure 2a’) and STEM‐mapping (Figure S8, Supporting Information) of Co and Pt confirm that the bright spot is Co nanoparticle. However, Pt atoms can be detected on the whole scan path and relatively aggregate at the edge of Co nanoparticle. After annealing at 900 °C for 1 h, the lattice distance of the metal particle increases to 0.219 nm (belonging to CoPt (111) facet, Figure 2b).^[^ ^24^ ^]^ Compared with CoZ‐60Pt‐soak, there are fewer bright dots in the high‐resolution HAADF‐STEM image of CoZ‐60Pt, indicating the migration of Pt atoms from carbon substrate to Co nanoparticle under annealing. The STEM linear analysis (Figure 2b’) indicates the transformation from Co nanoparticle to CoPt nanoalloy, which is proved by the X‐ray diffraction (XRD) pattern in **Figure** **3**a. To show the CoPt signal clearly, the scanning rate of XRD measurements was reduced to 0.005 deg s^−1^. In Figure S9 (Supporting Information), a broad peak is obtained at 41.4°, which is attributed to (111) facet of CoPt (JCPDS card: no. 43–1358). Compared with pure Pt and Pt_3_Co, the peak of CoPt shows an obvious shift to a higher degree because of the doping of smaller atomic size of Co and compaction of crystal structure.^[^ ^25^ ^]^ The chemical state change of Pt elements before and after annealing was investigated by XPS. In Figure 3b, the percentage of Pt^0^ increases from 2.0% for CoZ‐60Pt‐soak to 64.5% for CoZ‐60Pt. Simultaneously, the percentage of Pt^4+^ decreases from 35.4% to almost zero. The TEM and XPS analyses indicate a significant change in the composition and chemical state of Pt‐based nanoparticles. However, the annealing process shows no significant influence on the chemical states of N and Co elements, as shown in Figure S4 (Supporting Information). As is well‐known, the high‐temperature annealing process often leads to serious agglomeration of metal nanoparticles. Here, two different substrates are compared to illustrate the space confinement effect of the graphene shell for ultrafine metal particles. As a control group, the porous carbon substrate was used to load Pt‐based nanoparticles (denoted as CoZ‐60Pt‐agglomeration). After annealing, large metal particles (Figure 2d,d’, MPS: 6.07 nm) are unevenly distributed on the carbon substrate. In the high‐resolution TEM images (Figure S10, Supporting Information), no graphene shell is observed on the metal particles. In contrast, the graphene shell with some defects and nanopores formed around Co nanoparticles during the pyrolysis of ZIF‐67, effectively limits the coalescence and growth of metal particles, leading to the smaller CoPt nanoparticles (Figure 2c,c’, MPS: 2.61 nm) after annealing process.

Based on the physicochemical characterization, the proposed formation mechanism of ultrafine CoPt nanoalloy follows four steps and is shown in Figure 3e. 1) Due to the space confinement of SiO_2_ shell, ZIF particles are isolated and the migration of Co atoms is restricted, leading to small Co nanoparticle, which further serves as the substrate for CoPt nanoalloy. 2) A lot of pores and defects formed by H_2_SO_4_ etching can provide abundant sites for Pt^4+^ adsorption. 3) During the migration of Pt atoms and transformation of metal nanoparticle under 900 °C annealing, the surrounding porous graphene shell protects nanoparticles from agglomeration by the space‐confined effect. 4) After annealing, the CoPt nanoalloy with Pt‐rich shells are formed spontaneously, which has high ORR activity.^[^ ^12^ ^]^ As shown in Figure 3c, compared with CoZ‐60Pt, the *E*_1/2_ of CoZ‐60Pt‐soak and CoZ‐60Pt‐agglomeration respectively decreases by about 227 and 171 mV, indicating the outstanding catalytic activity of CoPt nanoalloy. DFT analysis further reveals the reason for this high activity. Three kinds of active sites, Pt (111), CoPt@Pt (111), and Co@Pt (111), were constructed and geometry optimized. As shown in Figure S11 (Supporting Information), compared with the strain‐free Pt (111) surface, the distance of Pt−Pt decreases by 9.7% for Co@Pt (111) and 3.9% for CoPt@Pt (111). For Co@Pt (111), too short Pt−Pt distance hinders the adsorption of O_2_, as shown in Figure 3d and Table S1 (Supporting Information), leading to poor ORR performance. Inversely, an appropriate Pt−Pt distance can optimize the adsorption of O_2_, resulting in high ORR performance.^[^ ^6b^ ^]^

### 2.3 Active Site Control

**Figure 4:**
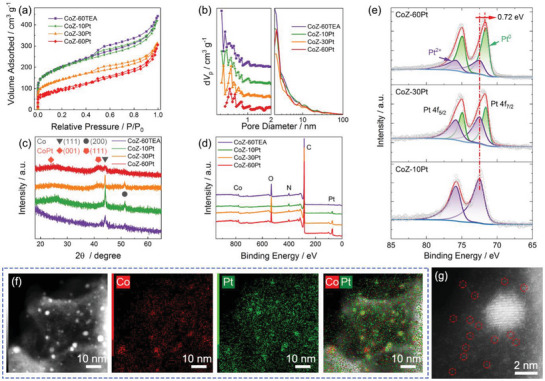
The a) nitrogen adsorption‐desorption isotherms and corresponding b) pore size distribution curves, c) XRD patterns, and d) XPS survey spectra of different catalysts with various Pt loading. e) The high‐resolution XPS spectra and corresponding deconvoluted curves of Pt 4f. f) The STEM‐EDS elements mapping of Co and Pt elements in CoZ‐10Pt. g) The HAADF‐STEM images of CoZ‐10Pt. The dotted circles indicate individual metal atoms.

To investigate the effective type of Pt‐based active site for ORR, the chemical state of the Pt element was finely controlled by adjusting the quantity of 2 mmol Pt^4+^ solution (10 mL for CoZ‐10Pt, 30 mL for CoZ‐30Pt and 60 mL for CoZ‐60Pt). With the increase in the amount of Pt, the metal content in the final catalyst increases obviously, as shown in thermogravimetric analysis (TGA, Figure S12a, Supporting Information). The accurate estimation of Co and Pt content was obtained from inductively coupled plasma optical emission spectrometry (ICP‐OES, Figure S12b, Supporting Information). In all three catalysts, the percentage of Co is relatively constant at ≈17 wt.%. However, the Pt content increases from 6 wt.% for CoZ‐10Pt to 15 wt.% for CoZ‐60Pt. Since the atomic ratio of Co and Pt in CoZ‐60Pt is 1, only 4.5 wt.% of Co atoms in catalysts are used to form CoPt nanoalloy. Therefore, more than 12.5 wt.% of Co is present as Co nanoparticles and Co‐N_x_ species. With the increase of Pt loading, the specific surface area decreases obviously from 712 m^2^ g^−1^ for CoZ‐60TEA to 331 m^2^ g^−1^ for CoZ‐60Pt (**Figure** **4**a and Figure S13, Supporting Information), due to the decrease in micropore volume by adsorption of Pt element (Figure 4b). As shown in XRD patterns (Figure 4c), the introduction of a small amount of Pt has no significant effect on the crystal structure of Co nanoparticles, indicating Pt atoms are preferably captured by defects and micropores to form atomically dispersed Pt metal sites. With the increase in Pt content, a diffraction peak of CoPt (111) facet appears at 41.4°. The XPS analysis provides direct evidence to identify the chemical state of Pt elements in different Pt‐content catalysts. In Figure 4d, the proportion of Pt on the catalyst surface gradually increases from 0.48 at% for CoZ‐10Pt to 0.81 at% for CoZ‐60Pt. The high‐resolution XPS spectra of Pt 4f indicate the evolution of Pt element (Figure 4e). For the CoZ‐10Pt, almost all Pt atoms are positively charged, which is due to their coordination with N or O atoms.^[^ ^26^ ^]^ The atomically dispersed Pt atoms are also observed by STEM‐EDS mapping and HAADF‐STEM images. Figure 4f,g shows that a large number of Pt single atoms surround the Co nanoparticles. By increasing the quantity of PtCl_4_ solution, the ratio of Pt^0^/Pt^2+^ rapidly increases from 0 for CoZ‐10Pt to 0.77 for CoZ‐30Pt and finally to 1.81 for CoZ‐60Pt (**Table** **1**), further verifying the XRD results. As the active site of ORR, CoPt nanoalloy catalyst exhibits very high catalytic activity, confirmed by other experimental and theoretical research.^[^ ^6^, ^9^ ^]^

**Table 1:** The specific surface area and element content of different CoPt nanoalloy catalysts

Samples	BET area [m^2^ g^−1^]	XPS element content				Pt 4f		Pt^0^/Pt^2+^
		C [at%]	N [at%]	Co [at%]	Pt [at%]	Pt^2+^ [at%]	Pt^0^ [at%]	
CoZ‐60TEA	712	92.99	5.45	1.57	–	–	–	–
CoZ‐10Pt	685	92.84	5.85	0.87	0.43	100.00	0	0
CoZ‐30Pt	498	95.64	3.14	0.74	0.48	56.57	43.43	0.77
CoZ‐60Pt	331	94.60	3.52	1.07	0.81	35.54	64.46	1.81

### 2.4 Performance Tests

**Figure 5:**
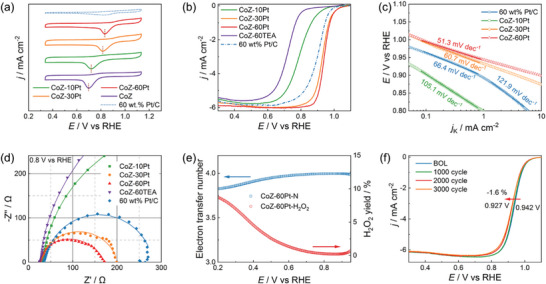
The ORR a) CV, b) LSV (1600 rpm) curves, and corresponding c) Tafel plots are shown as kinetic current densities (*j*_k_) normalized with RDE geometric area. d) The EIS curves of different catalysts at 0.8 V versus RHE by RDE test at 1600 rpm. e) Electron transfer number and H_2_O_2_ yield of CoZ‐60Pt catalyst in 0.1 m HClO_4_ in the range of 0.20‒0.95 V versus RHE. f) The LSV curves of CoZ‐60Pt catalyst after 0, 1000, 2000, and 3000 cyclic voltammetry scanning.

The obtained CoPt catalysts with different Pt‐based active sites were first examined by RDE in 0.1 _M_ HClO_4_ solution. As shown in **Figure** **5**a, cyclic voltammetry (CV) curves in oxygen‐saturated solution of different catalysts show obvious peaks in the potential range of 0.6 to 0.9 V versus RHE, corresponding to the ORR process. With the increase of Pt loading, the peak position of ORR gradually shifts to positive potential. During CV scanning in oxygen‐free solution (Figure S14, Supporting Information), CoZ‐10Pt exhibits the features of double‐layer capacitance due to the absence of metallic Pt (as discussed in Figure 4e). With the increase of Pt content, the electrochemical surface area (ECSA) increases from 47.8 m^2^ g^−1^ for CoZ‐30Pt to 58.1 m^2^ g^−1^ for CoZ‐60Pt. The linear sweep voltammetry (LSV) analysis provides a quantitative evaluation in Figure 5b. Compared to CoZ‐60TEA and CoZ‐10Pt, the LSV curve of CoZ‐30Pt dramatically shifts to positive potential, which is due to the formation of CoPt nanoalloy. With further increases in the Pt ratio, the *E*_1/2_ increases to 0.942 V versus RHE for CoZ‐60Pt. The MA and specific activity (SA) of CoZ‐60Pt can reach 525.9 mA mg_Pt_^−1^ and 1.55 mA cm_Pt_^−2^ at 0.9 V versus RHE, respectively, which is much larger than commercial 60 wt% Pt/C and CoZ‐30Pt (as shown in Figure S15 and Table S2). As shown in Tafel plot (Figure 5c), the similar curves of CoPt nanoalloy and Pt‐based catalysts suggest the similar rate‐determining step (RDS, formation of M−OOH).^[^ ^27^ ^]^ Furthermore, the Tafel slope of CoZ‐60Pt (51.3 mV dec^−1^) is smaller than those of CoZ‐30Pt (60.7 mV dec^−1^) and commercial 60 wt% Pt/C (66.4 mV dec^−1^) indicating the fast ORR kinetics.^[^ ^28^ ^]^ It is noteworthy that commercial 60 wt.% Pt/C has two Tafel regions with slopes close to 66.4 and 121.9 mV dec^−1^ at low and high overpotentials, respectively, which are attributed to differences in adsorbate coverage of oxygen‐containing species.^[^ ^29^ ^]^ However, both CoZ‐30Pt and CoZ‐60Pt show only one Tafel region, implying the anion adsorption effects on CoPt nanoalloy may be inhibited,^[^ ^30^ ^]^ which is consistent with the DFT analysis. The electrochemical impedance spectroscopy (EIS, Figure 5d) curves at high potential (0.8 V versus RHE) further confirms the highest ORR performance of CoZ‐60Pt catalyst with the smallest charge transfer resistance. During the rotating ring‐disk electrode (RRDE, Figure S16, Supporting Information) evaluation, in the wide potential range of 0.65‒0.95 V versus RHE, the electron transfer number of CoZ‐60Pt is above 3.99, while the H_2_O_2_ yield is lower than 1.00% (Figure 5e), indicating the ORR on CoPt nanoalloy prefers to follow 4‐electron pathway. With the decrease of applied potential, 2‐electron pathway is more pronounced,^[^ ^31^ ^]^ while the electron transfer number is still larger than 3.83. To investigate the stability in acid environment, accelerated degradation test was conducted from 0.75 to 1.10 V versus RHE. As shown in Figure 5f, the *E*_1/2_ decreases from 0.942 V versus RHE for the beginning of life (BOL) to 0.927 V versus RHE after 3000 CV cycles. Compared with BOL, the ratio of Pt/Co increases from 1.02 to 1.18 at the end of life (EOL) (Figure S17, Supporting Information), which is caused by the removal of the unstable Co atoms. The element mapping analysis of CoPt nanoalloy at EOL in Figure S18 (Supporting Information) reflects that the distribution of Co and Pt atoms also overlap, indicating the robust CoPt structure in the acidic environment. The high stability of CoZ‐60Pt can be attributed to the alloying of Co and Pt atoms, which enhances the Co(3d)‒Pt(5d) orbital coupling along the crystallographic *c* direction.^[^ ^25^, ^32^ ^]^

**Figure 6:**
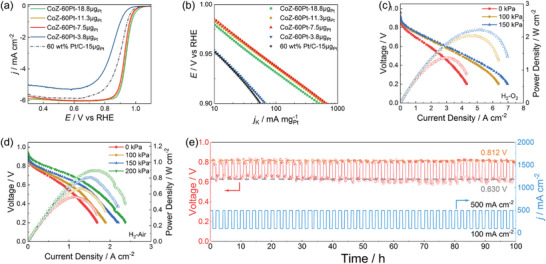
The a) ORR LSV curves and corresponding b) Tafel plots are given as kinetic current densities (*j*_k_) normalized with Pt loading by RDE at 1600 rpm. The PEMFC tests for CoZ‐60Pt with different backpressure at 80 °C of c) H_2_‐O_2_ and d) H_2_–air supplying. e) The stability curves of CoZ‐60Pt‐0.2 mg_Pt_ cm^−2^ at 80 °C with 50% relative humidity.

The catalytic performance of CoZ‐60Pt with different catalyst loading was explored by RDE. In **Figure** **6**a,b, even if the Pt loading on RDE (0.196 cm^2^) decreases to 3.8 µg, the *E*_1/2_ reaches as high as 0.852 V versus RHE, which is only 50 mV lower than Pt/C (Pt loading 15.0 µg). With the increase of Pt loading, the *E*_1/2_ of CoZ‐60Pt increases significantly. However, the *E*_1/2_ remains almost unchanged at around 0.93‒0.94 V versus RHE beyond 7.5 µg_Pt_, indicating that excess catalyst cannot further improve the ORR performance during RDE test. Relatively low Pt loading (CoZ‐60Pt‐7.5µg_Pt_) shows the highest MA of 681.8 mA mg_Pt_^−1^, which is also competitive in the Pt‐based ORR catalysts reported recently, as shown in Figure S19. Based on the RDE result, the CoZ‐60Pt catalyst was selected as a promising candidate for the cathode catalyst layer of PEMFC. As a comparison, the PEMFC performance of CoZ‐60TEA catalyst was also carried out under H_2_ and O_2_ supplying with 100 kPa backpressure. In Figure S20 (Supporting Information), the open‐circuit voltage (OCV) of CoZ‐60TEA‐based MEA is very low (< 0.75 V), even the catalyst loading increasing to 4.0 mg cm^−2^. After alloying with Pt element, the OCV and maximum power density increase dramatically, suggesting the excellent ORR performance of the CoPt nanoalloy catalyst, which is consistent with the RDE result. As a classic Pt‐based catalyst for commercial application, the MEA fabricated by 60 wt.% Pt/C (Johnson Matthey, 0.2 mg_Pt_ cm^−2^) exhibits high OCV at 0.911 V (Table S3, Supporting Information). Compared with Pt/C, the OCV of CoZ‐60Pt (0.2 mg_Pt_ cm^−2^) is much higher (0.960 V). The maximum power density of CoZ‐60Pt reaches 2.04 W cm^−2^, which is 37% higher than that of Pt/C (Figure S21, Supporting Information). Furthermore, the maximum power density of CoZ‐60Pt can be improved to 2.22 W cm^−2^ under H_2_ and O_2_ supplying with 150 kPa backpressure (Figure 6c). In order to verify the performance of this catalyst under actual fuel cell conditions, air was supplied into the cathode catalyst layer to replace oxygen. As shown in Figure 6d, the maximum power density of CoZ‐60Pt reaches 0.923 W cm^−2^ under 200 kPa backpressure. Meanwhile, the limiting current density reaches 2.45 A cm^−2^ at 0.2 V, further confirming the excellent catalytic performance of this catalyst in PEMFC. To investigate the stability of CoPt‐based MEA, dynamic pulse current tests between 100 and 500 mA cm^−2^ were carried out at 80 °C and 50% relative humidity, as shown in Figure 6e. Although the voltage fluctuates slightly during the test, it is constant at 0.812 V for 100 mA cm^−2^ and 0.630 V for 500 mA cm^−2^, reflecting the outstanding stability, which is consistent with the RDE test. The excellent activity and stability of the CoPt nanoalloy catalyst can be attributed to two aspects. 1) Pt atoms preferentially occupy the under‐coordinated surface site during the annealing at 900 °C, thus several layers of Pt atoms spontaneously coat on the nanoalloy core.^[^ ^33^ ^]^ Most Co atoms do not directly contact with the acidic environment, leading to the good stability. The DFT result indicating the strain effect of CoPt nanoalloy core will improve the ORR performance of Pt surface layer. 2) The porous graphene shell formed during the pyrolysis of ZIF‐67 also provides a protective shell and inhibits metal aggregation and loss during both the catalyst synthesis and dynamic operation. By a combination of a robust CoPt nanoalloy structure and protective graphene shell, the CoZ‐60Pt catalyst exhibits advanced ORR performance with remarkable potential for PEMFC application.

## 3 Conclusion

High performance and low Pt loading are major goals for PEMFC research. This work develops a two‐step space confinement method for synthesizing ultrafine CoPt nanoalloy catalyst for reliable fuel cell performance. The nanoalloy exhibited high ORR performance during RDE and PEMFC tests and outstanding stability under dynamic conditions. During the RDE tests, the MA of CoZ‐60Pt reached 681.8 mA mg_Pt_^−1^ (at 0.9 V versus RHE) with only 7.5 µg_Pt_ loaded on 0.196 cm^2^ electrode, which was almost 10 times than that of commercial Pt/C. PEMFC tests showed a high performance of this catalyst (0.2 mg_Pt_ cm^−2^) with 1757 mA mg_Pt_^−1^ @ 0.8 V and 2.22 W cm^−2^ maximum power density. Even after 3000 CV tests or 100 h dynamic pulse current tests, CoZ‐60Pt catalyst showed excellent stability. We attribute this to the following three aspects. 1) Due to the ZIF‐67 modification and confinement of SiO_2_ shell, the MPS of Co particles can be controlled to below 3 nm, which provides an outstanding substrate for the formation of ultrafine CoPt nanoalloy particles. 2) Due to the space confinement of porous graphene shell, which is in situ formed on Co particles during ZIF‐67 pyrolysis, the MPS of CoPt nanoalloy can be regulated at around 2.61 nm, resulting in a highly exposed surface as well as protection of the nanometal. 3) The CoPt nanoalloy particle with Pt‐rich shell can be obtained through the adsorption−annealing strategy, which further improves the catalytic activity and stability. Above all, the CoPt nanoalloy catalyst with outstanding ORR performance was obtained based on a ZIF‐67 derived carbon substrate, which presents a promising approach for CoPt‐based catalyst designs for PEMFC cathode materials.

## 4 Experimental Section

### Materials

Cobaltous nitrate hexahydrate (Co(NO_3_)_2_ 6H_2_O, 99%), 2‐methylimidazole (98%), polyvinyl pyrrolidone (PVP, K29‐32), hexadecyl trimethyl ammonium bromide (CTAB, 99%), tetraethyl orthosilicate (TEOS, 99%) and platinic chloride (PtCl_4_, 99.9%) were obtained from Aladdin, China. Triethylamine (99%) sodium hydroxide (NaOH, 97%), and concentrated sulfuric acid (H_2_SO_4_, 95%‐98%) were purchased from Sigma‐Aldrich, China. Methanol was purchased from Tianjin Yuanli, China. Ar, O_2_, N_2_, and H_2_ (99.999%) were supplied by Linde, China. All the materials were used as received.

### Synthesis of ZIF‐67@SiO_2_ Precursor

Similar to previous reports, ZIF‐67 was synthesized in methanol with Co^2+^ as a metal node and 2‐methylimidazole as an organic ligand. In detail, cobaltous nitrate hexahydrate (1.164 g, 4 mmol) and polyvinyl pyrrolidone (0.5 g) were dissolved in methanol (100 mL), denoted as solution A. The solution B was prepared from a mixture of methanol (100 mL), 2‐methylimidazole (1.314 g, 16 mmol), and triethylamine (0, 40, 50, 60, 70, or 80 µL). Solution A and B were mixed at room temperature for 24 h using a magnetic stirrer at 300 rpm. The resulting purple powder was collected by centrifugation and washed with methanol twice. Then, ZIF‐67 was re‐dispersed in methanol (100 mL) for 10 min in an ultrasonic bath. A solution of water (55 mL), CTAB (0.25 g) and 2‐methylimidazole (5.00 g) was next added into above mixture, followed by magnetic stirring at 400 rpm. After 15 min, TEOS (1 mL) was added into the above mixture dropwise and kept stirring for 1.5 h. Finally, the ZIF‐*x*TEA@SiO_2_ core–shell nanoparticle was recovered by centrifugation (*x* is the added amount of TEA).

### Fabrication of ZIF‐67‐Derived Co‐N‐C Substrate

The obtained ZIF‐67@SiO_2_ was pyrolyzed at 700 °C for 2 h under the protection of Ar (200 mL min^−1^). After ambient cooling to room temperature, the powder was transferred into 40 mL NaOH solution (4 g NaOH in 40 mL water). The mixture was heated to 50 °C and kept stirring for 24 h to dissolve the SiO_2_ shell. Then, it was further stirred in 0.5 M H_2_SO_4_ at 80 °C for 6 h. Subsequently, the Co‒N‒C substrate was collected by centrifugation and washed twice with water. The powder was further annealed at 900 °C for 1 h under the protection of Ar to obtain the final Co‒N‒C catalyst, denoted as CoZ‐*x*TEA (*x* is the added amount of TEA).

### Fabrication of CoPt Nanoalloy Catalyst

Before annealing in the above step, the obtained CoZ‐60TEA substrate (120 mg) was stirred in 60 mL PtCl_4_ solution (2 mmol in water) for 48 h. After filtering and drying, CoZ‐60Pt‐soak was obtained. The excess Pt precursor in the filtrate can be reused in the subsequent synthesis process. To form ordered CoPt alloy, it was annealed at 900 °C for 1 h under the protection of Ar (denoted as CoZ‐60Pt). The ratio of Co and Pt was controlled by adjusting the amount of PtCl_4_ solution. As the control samples, CoZ‐10Pt and CoZ‐30Pt with lower Pt content were fabricated from 10 or 30 mL PtCl_4_ solution, respectively.

### Characterization

Field emission transmission electron microscope (FE‐TEM) images were obtained on JEOL‐F200 FE‐TEM instrument, and the relative amount as well as distribution of Co, Pt, C, and N elements were detected by an energy dispersive X‐ray detector attached to FE‐TEM. X‐ray diffraction (XRD) patterns of obtained ZIF precursor and electrode were acquired on a diffractometer (D8‐Focus) using the Cu K*α* radiation (k = 0.15418). X‐ray photoelectron spectra (XPS) patterns were recorded using an X‐ray Photoelectron Spectrometer (Thermo ESCALAB 250XI) with an aluminum (mono) K*α* source (1486.6 eV). Inductively coupled plasma (ICP) was conducted by a Shimadzu ICPE‐9800 (OES).

### RDE Test

The electrochemical measurements were carried out on an Autolab 302N potentiostat/galvanostat with a standard three‐electrode system. A graphite rod, a saturated calomel electrode (SCE), and a glassy carbon RDE/RRDE (Pine Research Instrumentation) coated with catalysts film served as the counter electrode, reference electrode, and working electrode, respectively. The ink was prepared with 5 mg catalyst ultrasonically dispersed in 1 mL mixture of Nafion (5 wt.%, 40 µL), isopropanol (730 µL), and ultrapure water (Milli‐Q Advantage A10, 230 µL). Then, the homogeneous ink was transferred to the glassy carbon surface (0.196 cm^2^) and dried naturally. Before linear sweep voltammetry (LSV), all electrodes were activated by cyclic voltammetry (CV) with at different scan rates (100, 50 and 10 mV s^−1^). For the LSV tests, the rotational speed of the working electrode was fixed at 1600 rpm and the sweep rate was 10 mV s^−1^ from negative to positive potential. The electron transfer number (n) and H_2_O_2_ yield (H_2_O_2_%) of the ORR process can be evaluated from RRDE data by the following equations.^[^ ^34^ ^]^

(1) $$n=\frac{4I_{D}}{I_{D}+\frac{I_{R}}{N}}$$

(2) $$H_{2}O_{2}\%=\frac{200I_{R}}{I_{R}+NI_{D}}$$

where *N* is the collection efficiency (*N* = 0.424), *I_R_* and *I_D_* are the ring current and disk current, respectively.

### PEMFC Test

For the PEMFC test, CoZ‐60Pt catalyst was ultrasonically dispersed in a mixture of Nafion ionomer (5 wt.%) and isopropanol/water (3:1 v/v) to obtain the cathode ink. To reduce the contact resistance between the catalyst layer and membrane, the ink was sprayed onto one side of a clean Gore 12 µm proton exchange membrane with an active area of 4 cm^2^ at 80 °C. A piece (2×2 cm^2^) of commercial gas diffusion electrode (60 wt.% Pt/C, 0.1 mg_Pt_ cm^−2^) was employed as the anode electrode. After that, it was assembled with the gas diffusion layer by hot‐pressing at 130 °C under 500 psi for 2 min to obtain the MEA. Then, the MEA was evaluated using LEANCAT‐PBT fuel cell test station. A constant gas flow of H_2_ (300 sccm)—O_2_ (500 sccm) or H_2_ (300 sccm)—air (1500 sccm) was applied to the anode and cathode, respectively, with 80% relative humidity at 80 °C and different backpressure. The durability test of CoPt‐based MEA with 0.2 mg_Pt_ cm^−2^ in the cathode was performed by a dynamic pulse current method between 100 and 500 mA cm^−2^ for 100 h with 50% relative humidity at 80 °C under H_2_‐air supplying. The repeating sequence was as follows: 100 mA cm^−2^ for 1 h, then 500 mA cm^−2^ for 1 h, for 50 cycles.

### DFT Calculations

All density functional theory (DFT) calculations were conducted by using DMol^3^ module.^[^ ^35^ ^]^ The generalized gradient approximation (GGA) with the Perdew‒Burke‒Ernzerhof (PBE) functional was used as the exchange‒correlation functional.^[^ ^36^ ^]^ A double numerical basis set plus polarization function (DNP) and DFT semi‐core pseudopotential (DSPP) were utilized for all the calculations. The k‐point grid was 3×3×1 for all calculations. A Fermi smearing method with a window size of 0.005 hartree was applied to the orbital occupancy to improve the computational performance. The convergence tolerances for the energy change, max force, and max displacement were 1 × 10^−5^ Ha, 0.002 Ha·Å^−1^ and 0.005 Å, respectively. A vacuum layer of 15 Å was chosen to avoid the interaction from adjacent periodical images.

## Conflict of Interest

The authors declare no competing interests.

## Author Contributions

W.Z. and J.Z. conceived the study and designed the experiments. J.Z., Y.Y., M.D.G., and W.Z. wrote the manuscript. W.Z., Y.P., H.L., and S.L. carried out the experiments and collected the data. W.Z. and S.L. prepared the data graphs. J.Z., Y.Y., X.L., H.L., R.Y., and M.D.G. discussed the results. All authors commented on the manuscript.

## Supporting information

Supporting Information

## Data Availability

The data that support the findings of this study are available from the corresponding author upon reasonable request.
