# Supplementary material for: Space Confinement to Regulate Ultrafine CoPt Nanoalloy for Reliable Oxygen Reduction Reaction Catalyst in PEMFC
Source: Adv Sci (Weinh). 2023 May 10;10(19):2206062. doi: 10.1002/advs.202206062 (PMC10323636; doi:10.1002/advs.202206062)
Supplement: Supplementary file 1 — Supporting Information [file ADVS-10-2206062-s001.pdf]

ADVANCED SCIENCE
Open Access

Supporting Information

for *Adv. Sci.*, DOI 10.1002/advs.202206062

Space Confinement to Regulate Ultrafine CoPt Nanoalloy for Reliable Oxygen Reduction Reaction Catalyst in PEMFC

Weikang Zhu, Yabiao Pei, Haotian Liu, Runfei Yue, Shilin Ling, Junfeng Zhang, Xin Liu, Yan Yin* and Michael D. Guiver*

Table of Contents

Contents	Page
**Figure S1.** The (a) The TEM images of ZIF-60TEA@SiO_2. (b) HAADF-STEM image and (c) N, (d) Co and (e) Si elemental mapping. (f) The overlapped image of Si and Co elements.	S1
**Figure S2.** The STEM-HAADF images of Co nanoparticle catalysts with (a) 0, (b) 40, (c) 50, (d) 60, (e) 70 and (f) 80 μL trimethylamine during ZIF synthesis.	S1
**Figure S3.** Histograms of particle size distribution for the Co nanoparticles prepared with different amounts of TEA.	S2
**Figure S4.** The high-resolution XPS spectra of (a) N 1s and (b) Co 2p for Co-based substrates; (c) N 1s and (d) Co 2p for CoPt-based catalysts.	S3
**Figure S5.** The (a) nitrogen adsorption–desorption isotherms and corresponding (b) pore size distribution curves of different substrates.	S3
**Figure S6.** The LSV curves of Co-based substrates prepared using different amounts of TEA.	S4
**Figure S7.** The element content in CoZ-60Pt catalyst by STEM-EDS.	S4
**Figure S8.** (a, c) STEM-HAADF image and corresponding (b, d) Co Pt elements mapping of (a, b) CoZ-60Pt-soak and (c, d) CoZ-60Pt.	S5
**Figure S9.** The XRD patterns of CoZ-60Pt catalyst under different scanning rates (top: 0.1 deg s^{-1}, below: 0.005 deg s^{-1}).	S5
**Figure S10.** The (a) TEM and (b, c) high-resolution TEM image of CoZ-60Pt-agglomeration.	S6
**Figure S11.** The atomic models of (a, d) Pt (111), (b, e) Co@Pt (111) and (c, f) CoPt@Pt (111) for DFT calculation. Green and yellow spheres represent Pt and Co atoms, respectively.	S6

**Figure S12.** (a) The TGA curves of different catalysts from 30 to 800 °C in air. (b) The Co and Pt content in catalyst is derived from the ICP-OES.	S7
**Figure S13.** BET surface area of different CoPt catalysts.	S7
**Figure S14.** The CV curves of different catalysts during RDE test in oxygen-free 0.1 M $HClO_4$ solution at 10 mV s^{-1}.	S8
**Figure S15.** Tafel plots are given as kinetic current densities (j_k) normalized with Pt loading by RDE at 1600 rpm in 0.1 M $HClO_4$.	S8
**Figure S16.** The LSV curves of CoZ-60Pt by RRDE evaluation.	S9
**Figure S17.** The EDS spectra and ratio of Co and Pt atoms for CoZ-60Pt catalyst before and after 3000 CV cycles.	S9
**Figure S18.** The (a) STEM-HAADF image and corresponding (b) N, (c) Co, (d) Pt elements mapping of CoPt-60Pt after 3000 CV cycles.	S10
**Figure S19.** A comparison of the CoZ-60Pt catalyst with other Pt-based catalysts in recent research. The dotted line represents the DOE target for Pt-based ORR catalyst.	S10
**Figure S20.** The PEMFC tests for CoZ with different catalyst loading at 80 °C under 100 kPa backpressure of H_2 and O_2 supply.	S11
**Figure S21.** The PEMFC tests for CoZ-60Pt and 60 wt.% Pt/C (JM) at 80 °C under 100 kPa backpressure of H_2 and O_2 supply.	S11
**Table S1.** The distance of Pt and O atoms derived from DFT simulation.	S12
**Table S2.** The RDE parameters of different catalysts.	S13
**Table S3.** The PEMFC parameters of the MEA with different backpressure.	S14

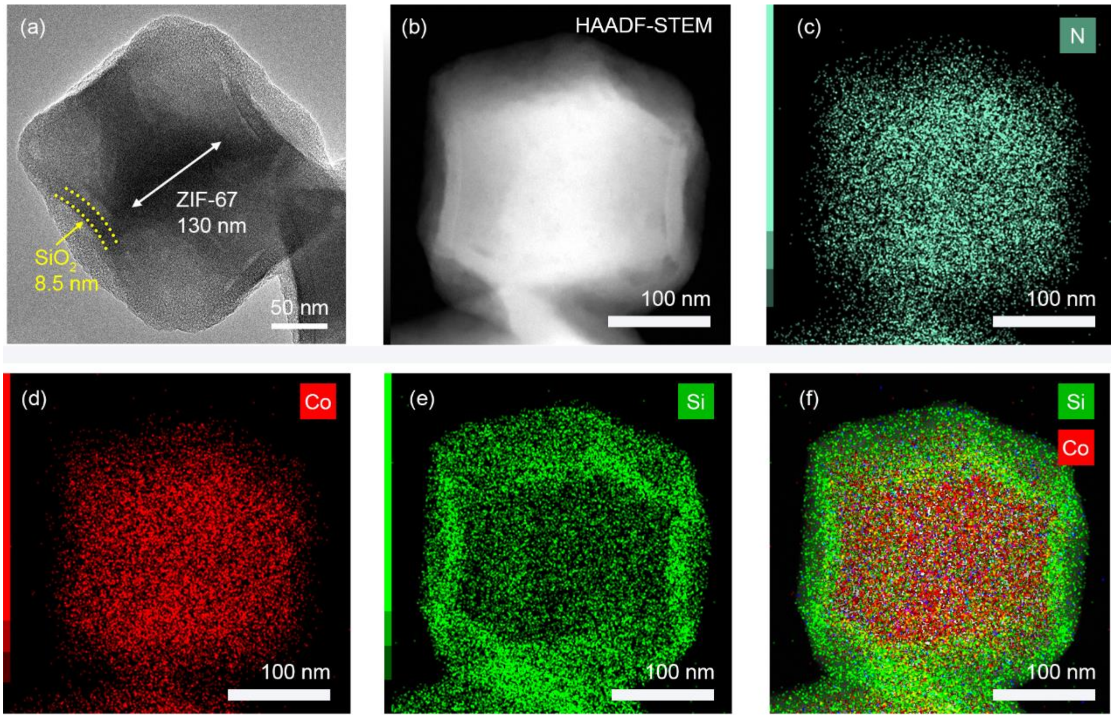

Figure S1. The (a) The TEM images of ZIF-60TEA@SiO_2. (b) HAADF-STEM image and (c) N, (d) Co and (e) Si elemental mapping. (f) The overlapped image of Si and Co elements.

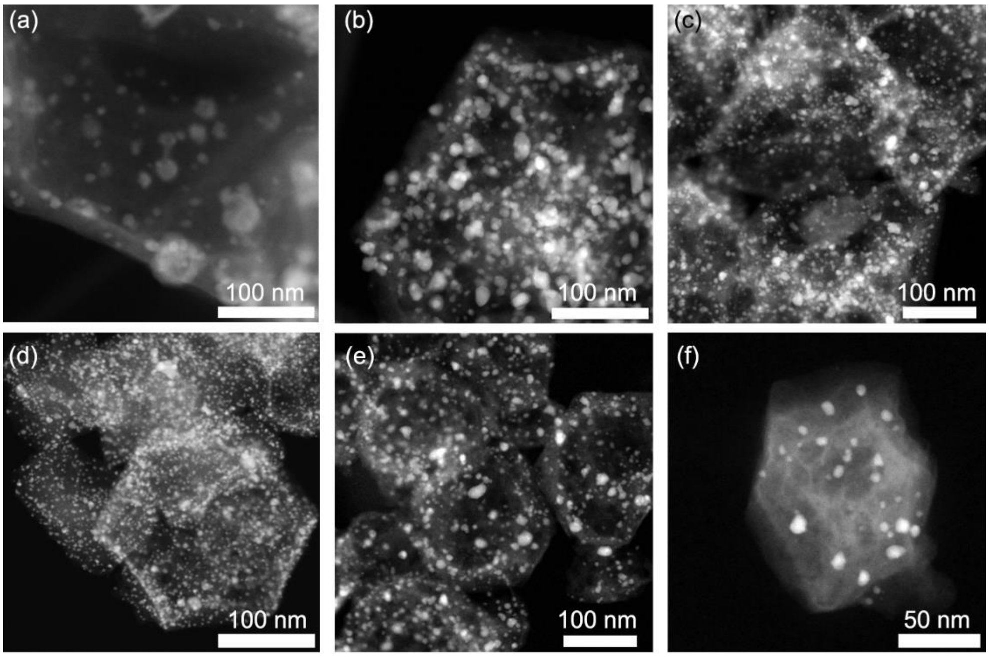

Figure S2. The STEM-HAADF images of Co nanoparticle catalysts with (a) 0, (b) 40, (c) 50, (d) 60, (e) 70 and (f) 80 μL trimethylamine during ZIF synthesis.

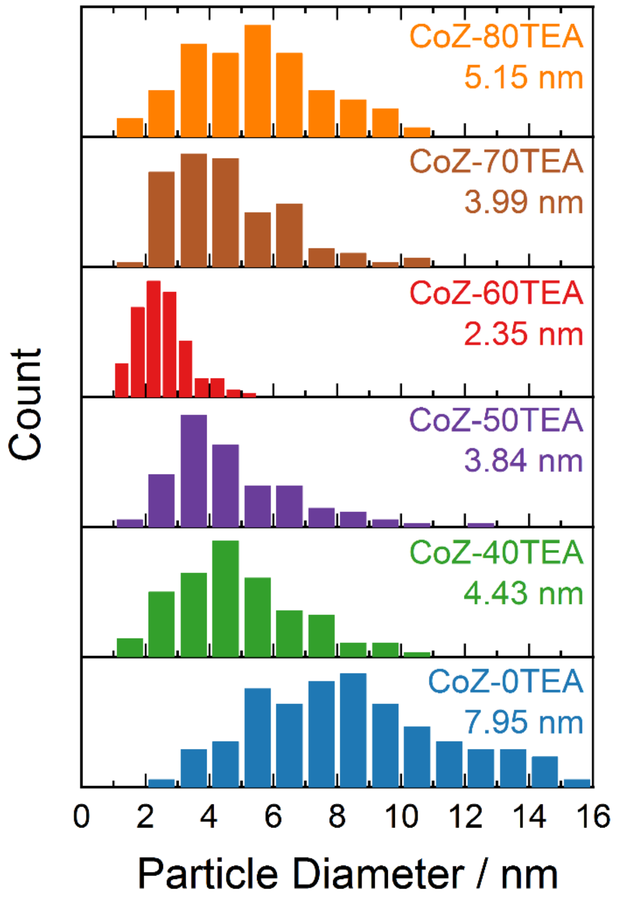

Figure S3. Histograms of particle size distribution for the Co nanoparticles prepared with different amounts of TEA.

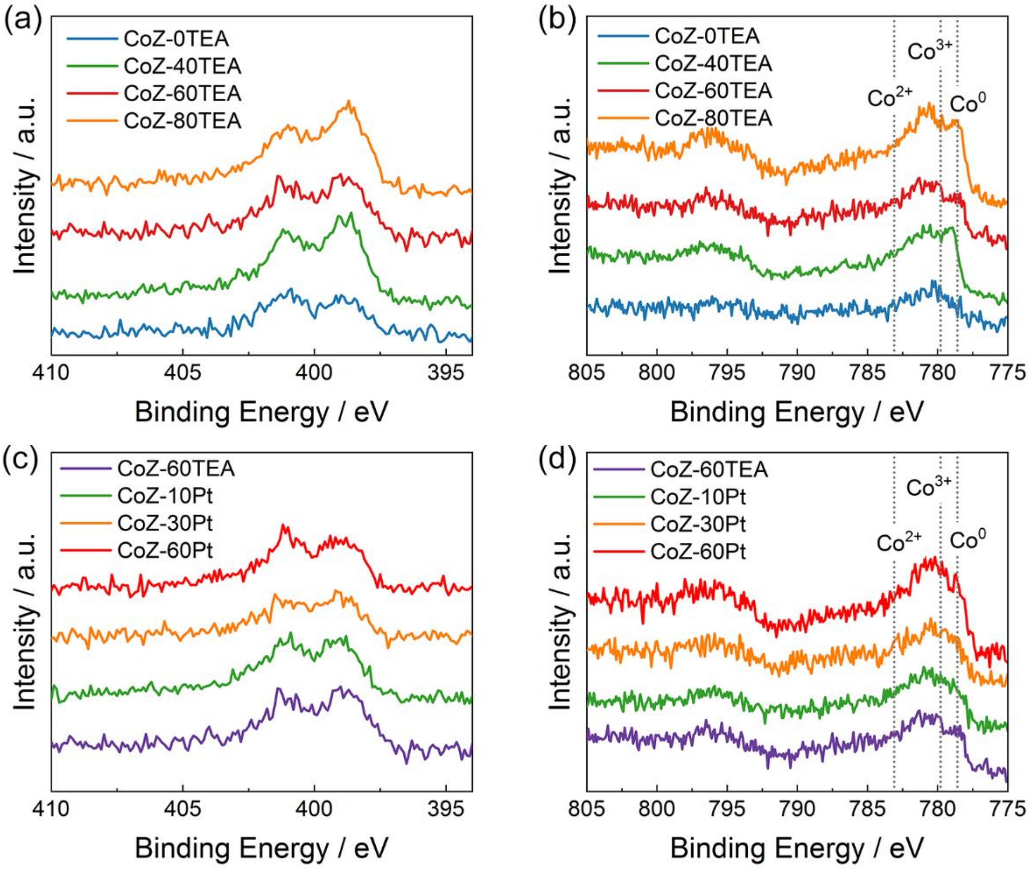

Figure S4. The high-resolution XPS spectra of (a) N 1s and (b) Co 2p for Co-based substrates; (c) N 1s and (d) Co 2p for CoPt-based catalysts.

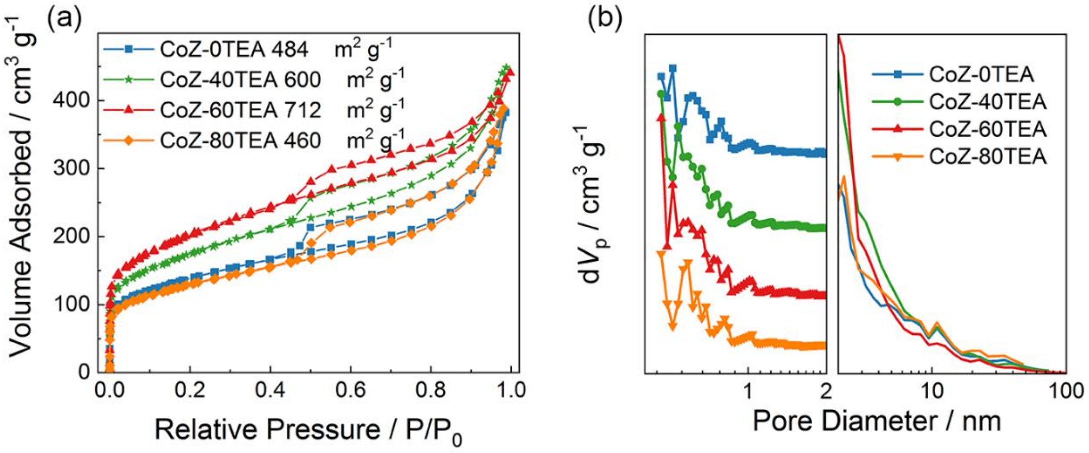

Figure S5. The (a) nitrogen adsorption–desorption isotherms and corresponding (b) pore size distribution curves of different substrates.

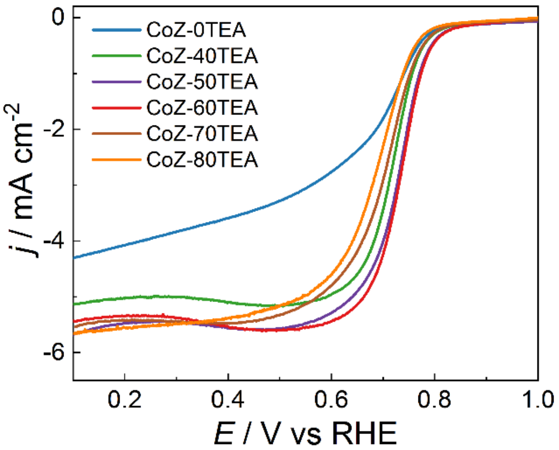

Figure S6. The LSV curves of Co-based substrates prepared using different amounts of TEA.

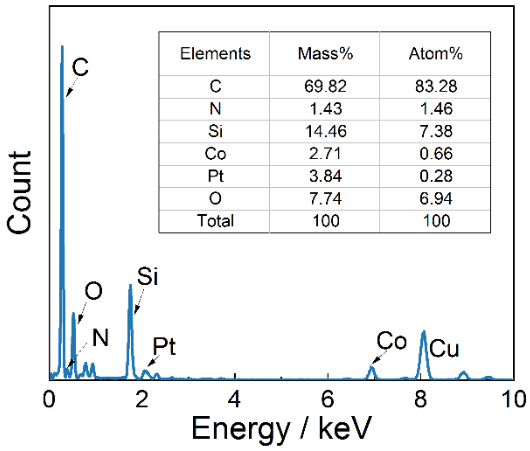

Figure S7. The element content in CoZ-60Pt catalyst by STEM-EDS.

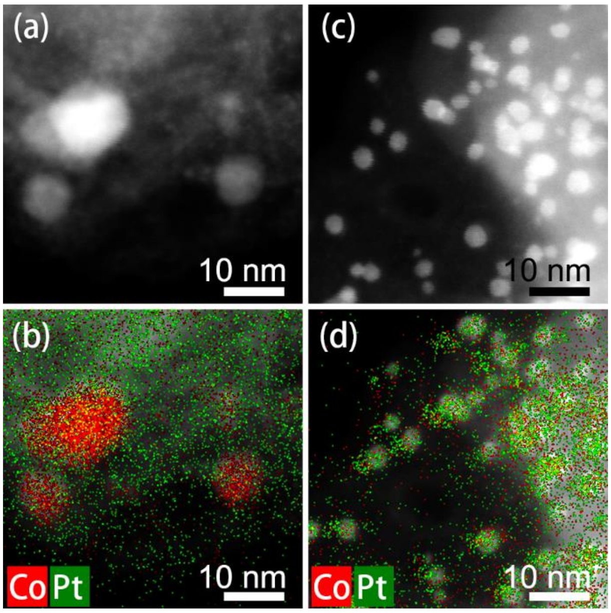

Figure S8. (a, c) STEM-HAADF image and corresponding (b, d) Co Pt elements mapping of (a, b) CoZ-60Pt-soak and (c, d) CoZ-60Pt.

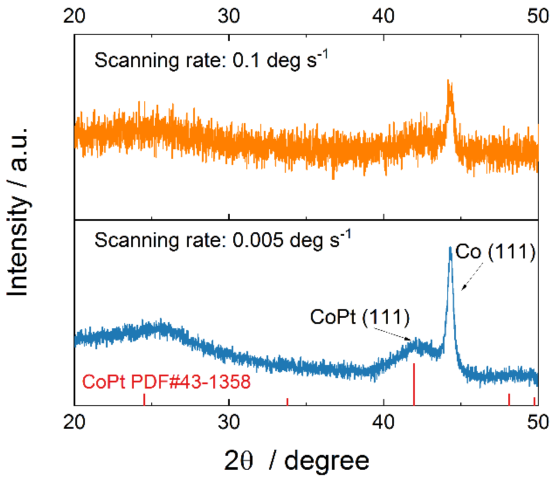

Figure S9. The XRD patterns of CoZ-60Pt catalyst under different scanning rates (top: 0.1 deg s^{-1}, below: 0.005 deg s^{-1}).

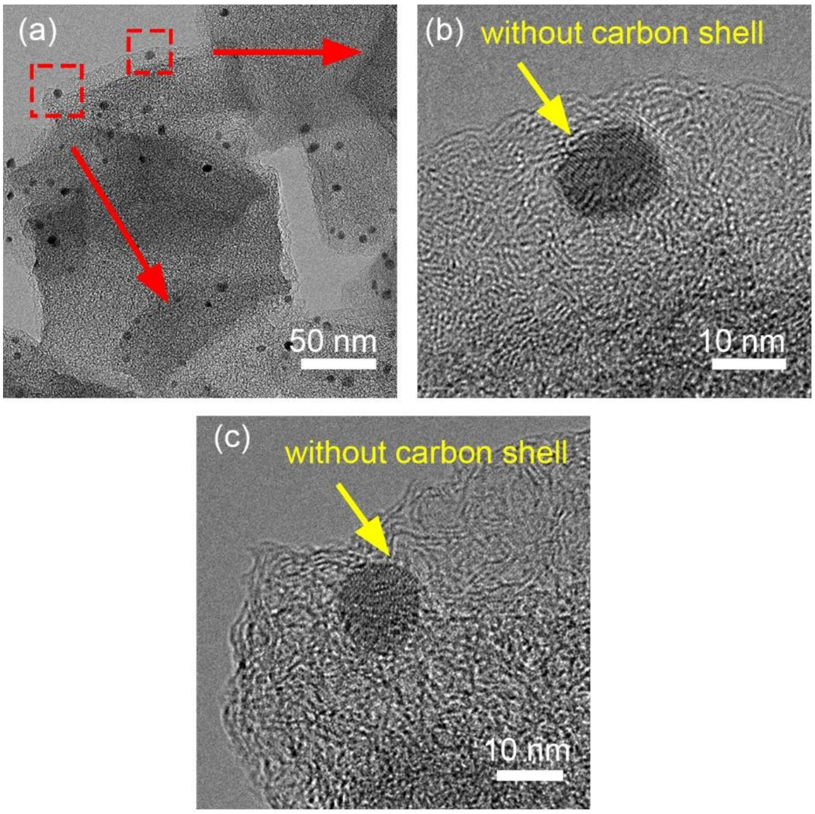

Figure S10. The (a) TEM and (b, c) high-resolution TEM image of CoZ-60Pt-agglomeration.

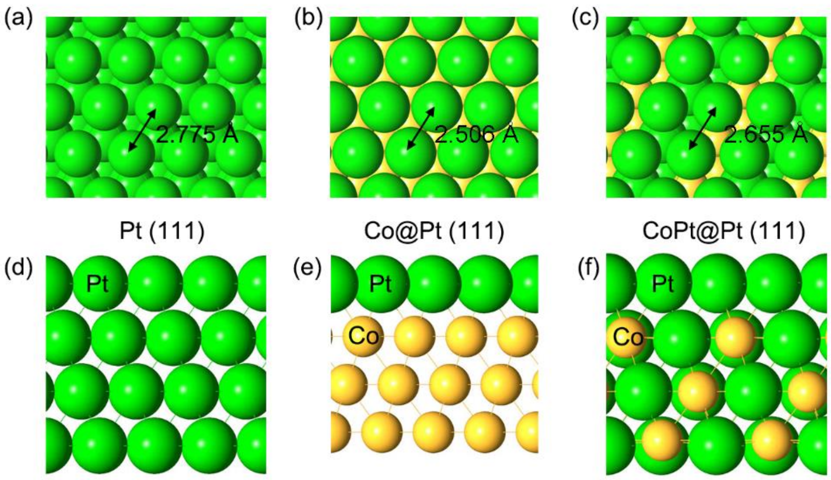

Figure S11. The atomic models of (a, d) Pt (111), (b, e) Co@Pt (111) and (c, f) CoPt@Pt (111) for DFT calculation. Green and yellow spheres represent Pt and Co atoms, respectively.

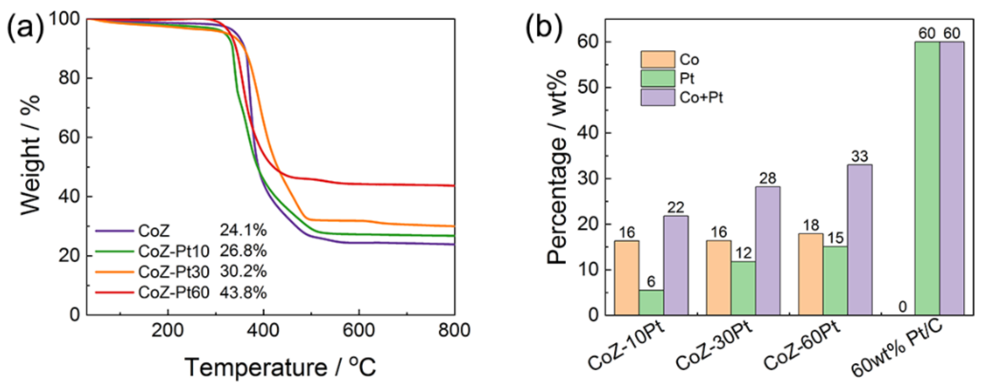

Figure S12. (a) The TGA curves of different catalysts from 30 to 800 °C in air. (b) The Co and Pt content in catalyst is derived from the ICP-OES.

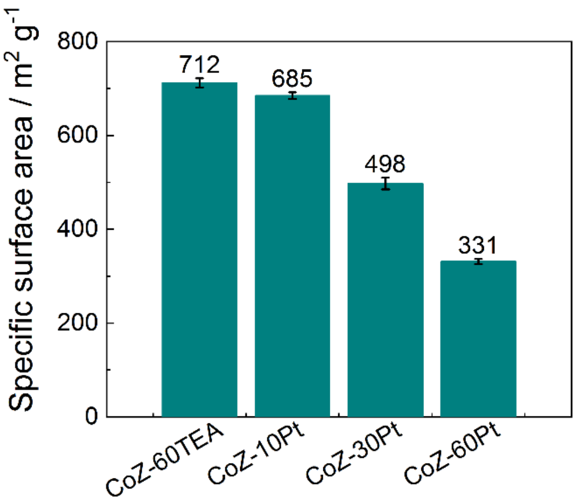

Figure S13. BET surface area of different CoPt catalysts.

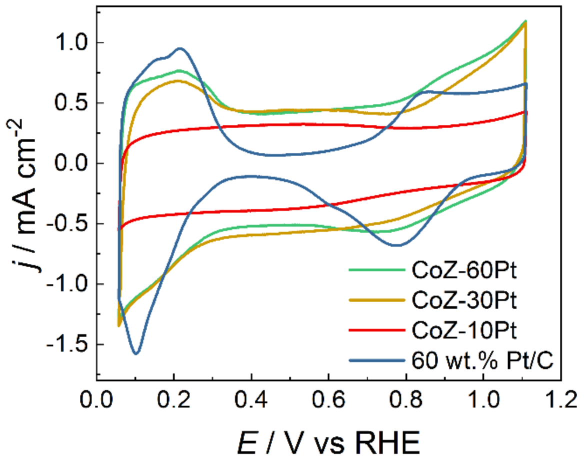

Figure S14. The CV curves of different catalysts during RDE test in oxygen-free 0.1 M $HClO_4$ solution at 10 mV s^{-1}.

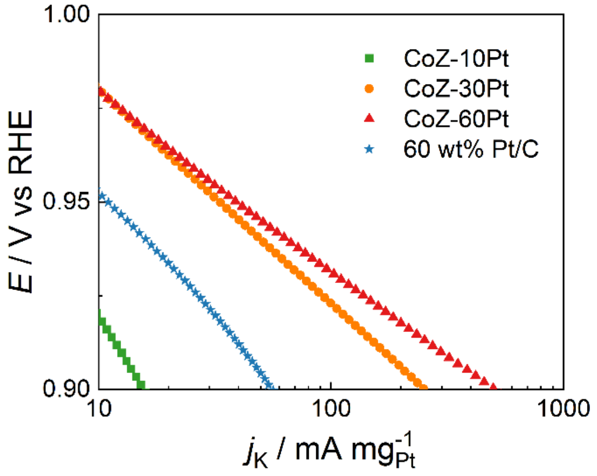

Figure S15. Tafel plots are given as kinetic current densities (j_k) normalized with Pt loading by RDE at 1600 rpm in 0.1 M $HClO_4$.

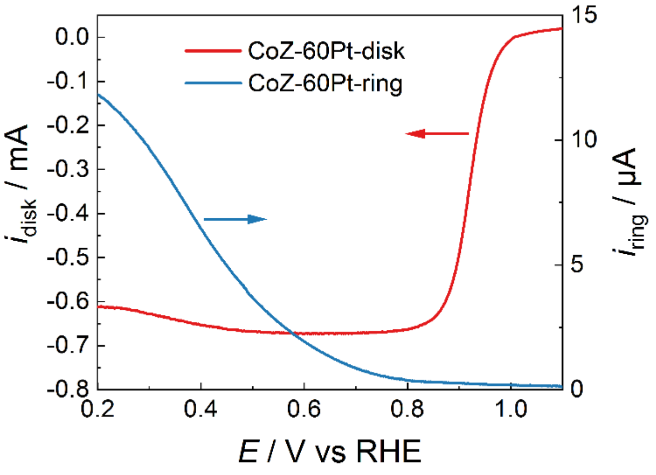

Figure S16. The LSV curves of CoZ-60Pt by RRDE evaluation.

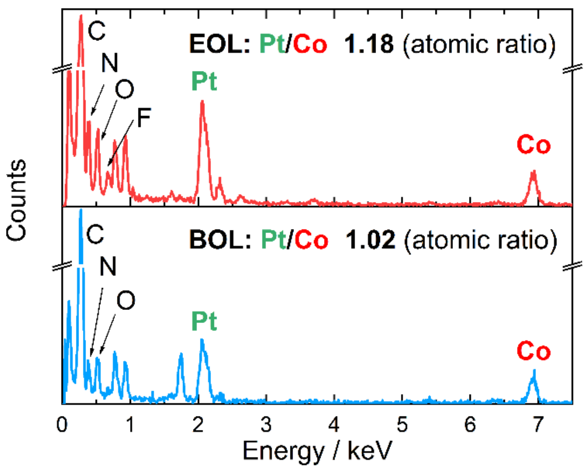

Figure S17. The EDS spectra and ratio of Co and Pt atoms for CoZ-60Pt catalyst before and after 3000 CV cycles.

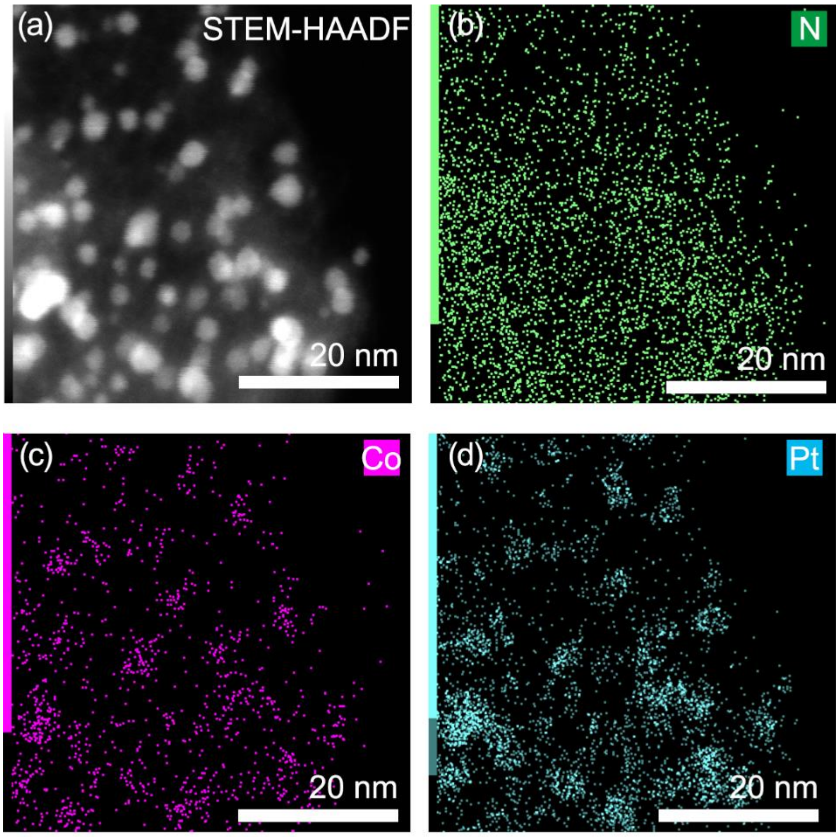

Figure S18. The (a) STEM-HAADF image and corresponding (b) N, (c) Co, (d) Pt elements mapping of CoPt-60Pt after 3000 CV cycles.

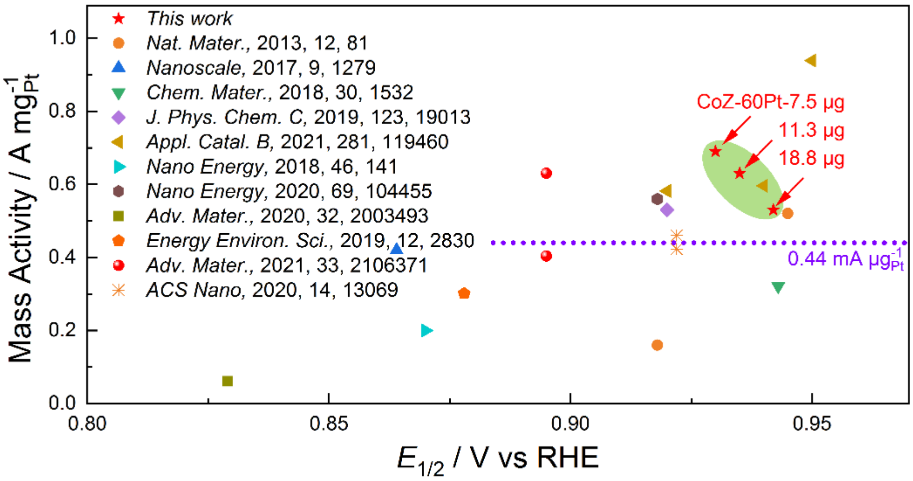

Figure S19. A comparison of the CoZ-60Pt catalyst with other Pt-based catalysts in recent research. The dotted line represents the DOE target for Pt-based ORR catalyst.

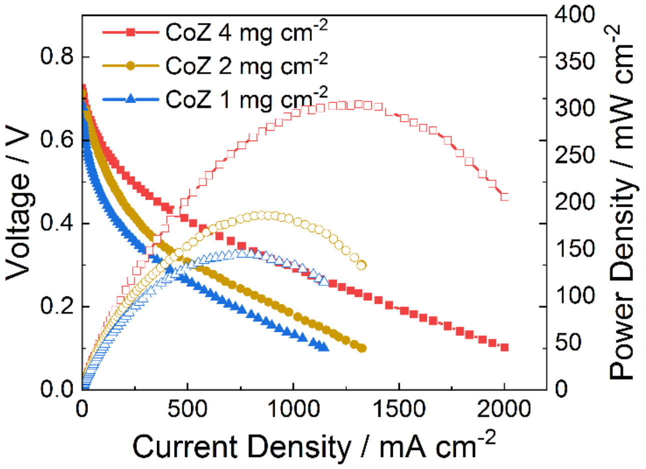

Figure S20. The PEMFC tests for CoZ with different catalyst loading at 80 °C under 100 kPa backpressure of H_2 and O_2 supply.

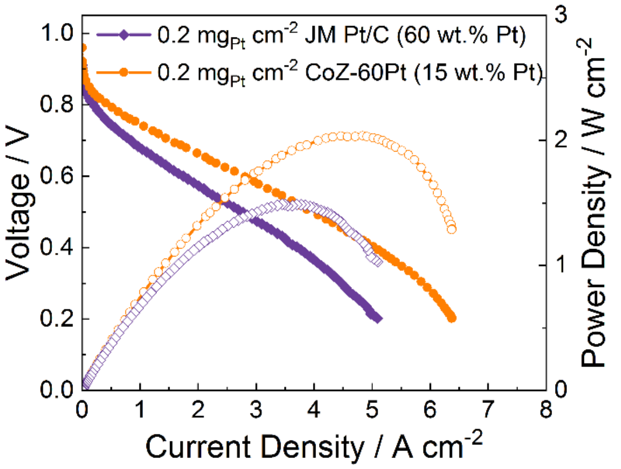

Figure S21. The PEMFC tests for CoZ-60Pt and 60 wt.% Pt/C (JM) at 80 °C under 100 kPa backpressure of H_2 and O_2 supply.

Table S1. The distance of Pt and O atoms derived from DFT simulation.

Sample	$d_{Pt\text{–}Pt}$ / Å	Strain / %	$d_{Pt\text{–}O}$ / Å	$d_{Pt\text{–}O}$ / Å
Pt (111)	2.775	0	2.061	2.062
Co@Pt (111)	2.506	-9.7%	2.420	2.604
CoPt@Pt (111)	2.665	-3.9%	2.132	2.191

Table S2. The RDE parameters of different catalysts.

Sample	$E_{1/2}$ (V vs RHE)	MA (mA mg_{Pt}^{-1})	SA (mA cm^{-2})	ECSA ($m^2\ g_{Pt}^{-1}$)	
**CoZ-60TEA**	**0.724**	**N/A**	**N/A**	**N/A**	
**CoZ-10Pt**	**0.797**	**15.2**	**N/A**	**N/A**	
**CoZ-30Pt**	**0.931**	**259.4**	**0.97**	**47.8**	**This work**
**CoZ-60Pt**	**0.942**	**525.9**	**1.55**	**58.1**	
**60 wt.% Pt/C**	**0.902**	**56.4**	**0.11**	**52.2**	
PtCo-600	-	1040	~1.5	72.2	*J. Electroanalytical Chem.*, 2022, **922**, 116728
PtCo/C-400	0.825	-	-	66	*J. Power Sources*, 2015, **293**, 274–282
Pt5Co@HGS	-	490	0.61	84	
Pt3Co@HGS	-	690	0.72	101	*Adv. Energy Mater.*, 2017, **7**, 1700835
PtCo@HGS	-	970	0.92	112	
Pt-Co/MWCNT	0.857	227.4	0.38	59.3	
Pt-Co/3D HPG	0.850	207.3	0.31	66.8	*ACS Appl. Mater. Interfaces*, 2021, **13**, 34397–34409
Pt-Co/3D rHPGO	0.841	166.9	0.31	53.1	
Pt–Co–W	0.97	2250	3.41	65.9	*J. Mater. Chem. A*, 2018, **6**, 10700–10709
Pt–Co	0.91	530	0.80	66.6	
Co15-Pt	0.916	290	0.713	41	
Co10Zn5-Pt	0.935	670	0.796	84	*Adv. Energy Mater.*, 2022, **12**, 2201600
Co12Zn3-Pt	0.935	680	0.767	88	
fct-PtCo/C@ILs	-	1040	1.4	74.46	*Inter. J. Hydrogen Energy*, 2022, **47**, 6312–6322
fct-PtCo/C	-	470	0.59	80.52	

Note: MA and SA are calculated at 0.9 V vs RHE by RDE.

Table S3. The PEMFC parameters of the MEA with different backpressure.

ORR catalyst	Pt loading in cathode / mg cm^{-2}	Backpressure / kPa	E_{OCV} / V	$j_{0.8V}$ / mA cm^{-2}	$j_{0.8V}$ / mA mg$_{Pt}^{-1}$	P_{MAX} / W cm^{-2}
60 wt% Pt/C	0.2	100	0.911	215	717	1.49
CoZ-60Pt	0.2	0	0.953	288	960	1.37
CoZ-60Pt	0.2	100	0.960	517	1723	2.04
CoZ-60Pt	0.2	150	0.964	527	1757	2.22
CoZ-60TEA	4 (catalyst)	100	0.730	-	-	0.31
